# Peripheral immune response in the African green monkey model following Nipah-Malaysia virus exposure by intermediate-size particle aerosol

**DOI:** 10.1371/journal.pntd.0007454

**Published:** 2019-06-05

**Authors:** Abigail Lara, Yu Cong, Peter B. Jahrling, Mark Mednikov, Elena Postnikova, Shuiqing Yu, Vincent Munster, Michael R. Holbrook

**Affiliations:** 1 NIAID Integrated Research Facility, Ft. Detrick, Frederick, MD, United States of America; 2 Virus Ecology Unit, Laboratory of Virology, Rocky Mountain Laboratories, Hamilton, MT, United States of America; University of Texas Medical Branch, UNITED STATES

## Abstract

The ability to appropriately mimic human disease is critical for using animal models as a tool for understanding virus pathogenesis. In the case of Nipah virus (NiV), infection of humans appears to occur either through inhalation, contact with or consumption of infected material. In two of these circumstances, respiratory or sinusoidal exposure represents a likely route of infection. In this study, intermediate-size aerosol particles (~7 μm) of NiV-Malaysia were used to mimic potential routes of exposure by focusing viral deposition in the upper respiratory tract. Our previous report showed this route of exposure extended the disease course and a single animal survived the infection. Here, analysis of the peripheral immune response found minimal evidence of systemic inflammation and depletion of B cells during acute disease. However, the animal that survived infection developed an early IgM response with rapid development of neutralizing antibodies that likely afforded protection. The increase in NiV-specific antibodies correlated with an expansion of the B cell population in the survivor. Cell-mediated immunity was not clearly apparent in animals that succumbed during the acute phase of disease. However, CD4+ and CD8+ effector memory cells increased in the survivor with correlating increases in cytokines and chemokines associated with cell-mediated immunity. Interestingly, kinetic changes of the CD4+ and CD8^bright^ T cell populations over the course of acute disease were opposite from animals that succumbed to infection. In addition, increases in NK cells and basophils during convalescence of the surviving animal were also evident, with viral antigen found in NK cells. These data suggest that a systemic inflammatory response and “cytokine storm” are not major contributors to NiV-Malaysia pathogenesis in the AGM model using this exposure route. Further, these data demonstrate that regulation of cell-mediated immunity, in addition to rapid production of NiV specific antibodies, may be critical for surviving NiV infection.

## Author summary

Nipah virus (NiV) infection in Malaysia, Bangladesh and India has been correlated with severe respiratory and neurological disease that led to death in over 50% of known cases. In this study, we used a nonhuman primate model for NiV infection to evaluate the peripheral immune response to virus infection in an effort to identify aspects of the immune response that may be important for survival. An aerosol exposure that targeted virus deposition in sinuses and upper respiratory tract was used in an effort to mimic a probable human exposure route. Following exposure, five of six animals included in the study succumbed to the infection. The survivor developed a virus-specific antibody response and showed clear evidence of cell-mediated immunity. Interestingly, the rate of change in CD4+ and CD8^bright^ T cell populations in the survivor over the course of the acute disease, were the reverse of animals that succumbed to infection. These data suggest that rapid development of virus-specific adaptive immunity is critical for survival of NiV infection.

## Introduction

A comprehensive understanding of disease processes requires the use of a model that accurately recapitulates significant components of human disease. In this study, we continue efforts to develop the African green monkey (AGM) model of Nipah virus (NiV) infection. This work focused on examining the peripheral immune response induced by NiV infection following exposure to intermediate-size aerosol particles of the Malaysian isolate of NiV (NiV-M). In addition to evaluating immune responses during the acute phase of disease, an animal that survived exposure has provided the opportunity to characterize the acute and convalescent immune responses to NiV-M infection and to identify immune characteristics of the animal that may have provided it with a competitive advantage for survival.

Nipah virus is a zoonotic virus that is transmitted to humans and other animals through contact with, or consumption of, excreta from infected fruit bats (*Pteropus spp*.) or other infected animals. In the first recognized outbreak of NiV infection in Malaysia and Singapore in 1998, a large number of infected humans developed neurological disease while others developed a severe respiratory disease [1, 2]. In this outbreak, the typical route of exposure was associated with close contact with infected pigs suggesting a fomite/contact or respiratory droplet route of exposure [3, 4]. An outbreak of NiV infection was first reported in January 2004 in Bangladesh with an initial focus of 12 cases with unknown etiology, but interaction with bats was a suspected commonality [5]. In this initial outbreak, both neurological and respiratory disease symptoms were reported. In more recent outbreaks in Bangladesh and India, there is an apparent higher frequency of respiratory disease compared to cases seen in Malaysia, although neurological disease is still common [6]. Rather than proximity to pigs, in Bangladesh and India human infection has been associated with the consumption of unpasteurized date palm sap and limited human-to-human transmission [7–9]. To date there have been over 600 known cases of NiV infection with a case fatality rate over 50%.

Previous work has established the AGM as a model for NiV infection, however, in most of these studies the mortality rate was near 100% [10–13]. In the AGM model, neurological involvement was not a clear and consistent component of the disease, although making assessments in nonhuman primates in a biocontainment environment to determine subtle neurological changes is challenging. Previous work has documented systemic vasculitis as a significant aspect of the disease with hemorrhage and pulmonary edema identified by both pathological assessment and computed tomography [10, 11, 13, 14]. Despite limited overt indication of neurological disease in this model, we have shown that animals exposed to intermediate-size (~7 μm) particles of NiV-M developed brain lesions that were identified by magnetic resonance imaging and were reminiscent of previously reported lesions in humans [14–18].

Clinical and pre-clinical studies of NiV infection published to date have focused primarily on clinical and pathological assessments of disease progression. In this study, in order to gain a better understanding of the disease process, comprehensive immunological characterization of the peripheral immune response in infected animals was performed. Analyses included evaluation of the antibody response, systemic cytokine responses and temporal changes in peripheral immune cell populations of individual animals. The objective of these analyses was to determine if there were characteristics of NiV infection in the AGM model that could be exploited as potential targets for development of medical countermeasures. One of the animals challenged in this study became infected and developed disease but survived the acute infection and progressed into convalescence. Evaluation of immunological responses of this animal identified characteristics that were unique to this animal relative to others included in this study and may have provided a competitive advantage allowing this animal to survive. In addition, the survivor developed a classic antibody response and T cell response indicative of protective adaptive immunity. The studies presented here highlight not only the variability of the host response between outbred animals, but also the value of understanding the role host immunity plays in disease progression and the importance of appreciating host diversity in the ability to survive disease.

## Results

### Survival

In this study animals were exposed to doses of NiV-M ranging from 22–90 pfu in the low dose group (average particle size 7.16 μm) and 408–1197 pfu in the high dose groups (average particle size of 6.41 μm) [14]. All animals developed acute disease with no clear observational differences between the low and high dose groups. One of the six inoculated animals survived the infection and recovered with no evident sequelae. This animal received a dose of approximately 400 pfu of NiV. Clinical and pathological assessments and medical imaging of these animals can be found in a companion paper [14].

### Antibody response

**Fig 1:**
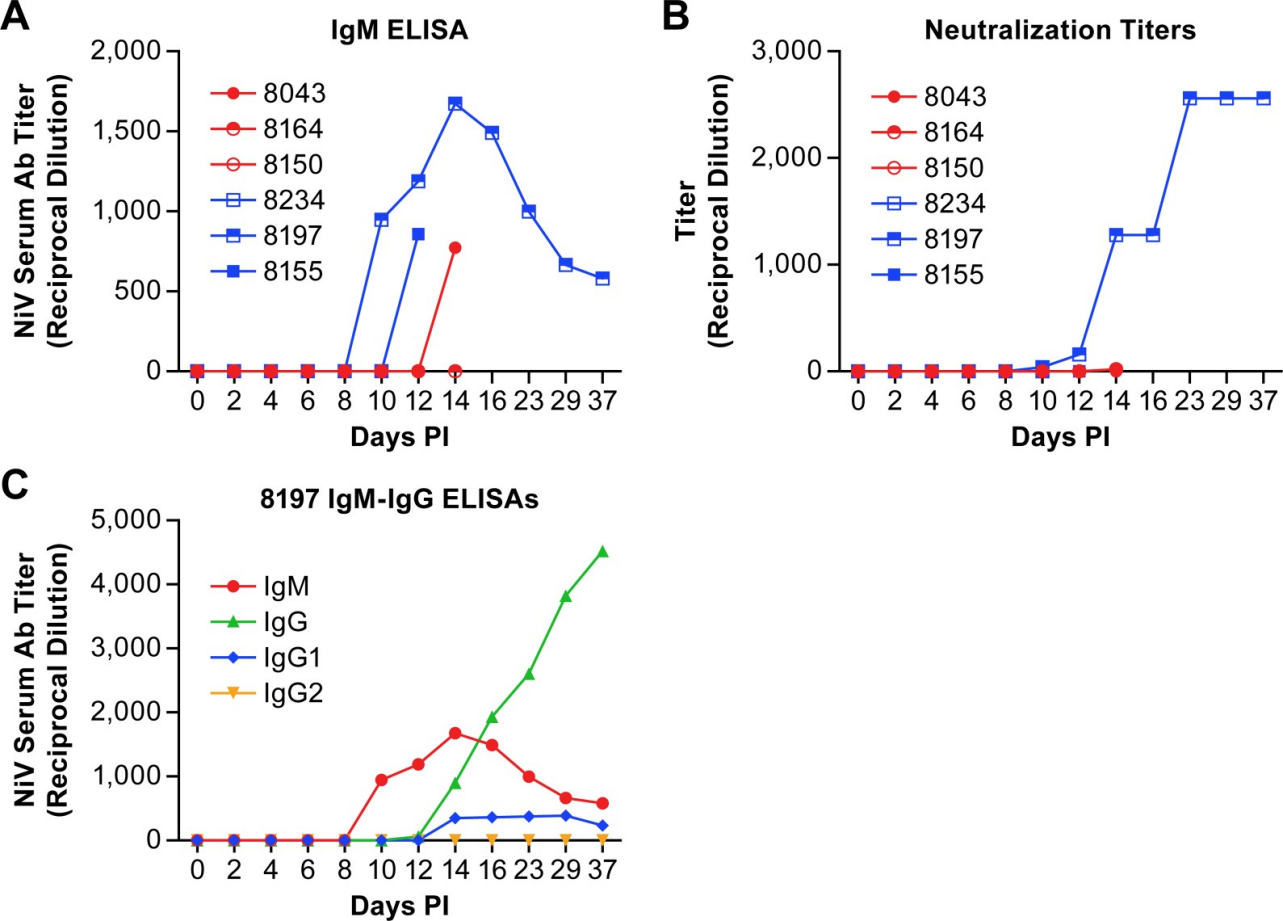
Antibody titers in plasma from AGMs infected with NiV. IgM (A) and neutralization titers (B) from individual animals infected with NiV over the course of disease. Animals in the low dose group are displayed as red circles and those in the high dose group as blue squares. IgM and IgG titers (C) from the animal that survived NiV infection.

Determination of the antibody response to NiV in infected AGMs found no antibody response until 10 dpi at which time one of the animals, the survivor (8197), had evidence of IgM and neutralizing antibody titers (Fig 1A and 1B). Two additional animals (8155 and 8043) developed IgM, but not neutralizing, titers on days 12 and 14, but ultimately succumbed to the infection. One animal (8164) had a very low neutralization titer (1:20) on day 14 post-infection. The IgM titers of 8197 peaked at 14 dpi when a NiV-specific IgG response became apparent with evidence of NiV-specific IgG1 antibodies, but not IgG2 antibodies (Fig 1C). IgG3 and IgG4 subclasses were not tested. The neutralizing antibody titer of 8197 plateaued at 23 dpi where it remained until the end of the study. None of the other animals in this study had apparent neutralizing antibody titers.

### Systemic cytokine response

**Fig 2:**
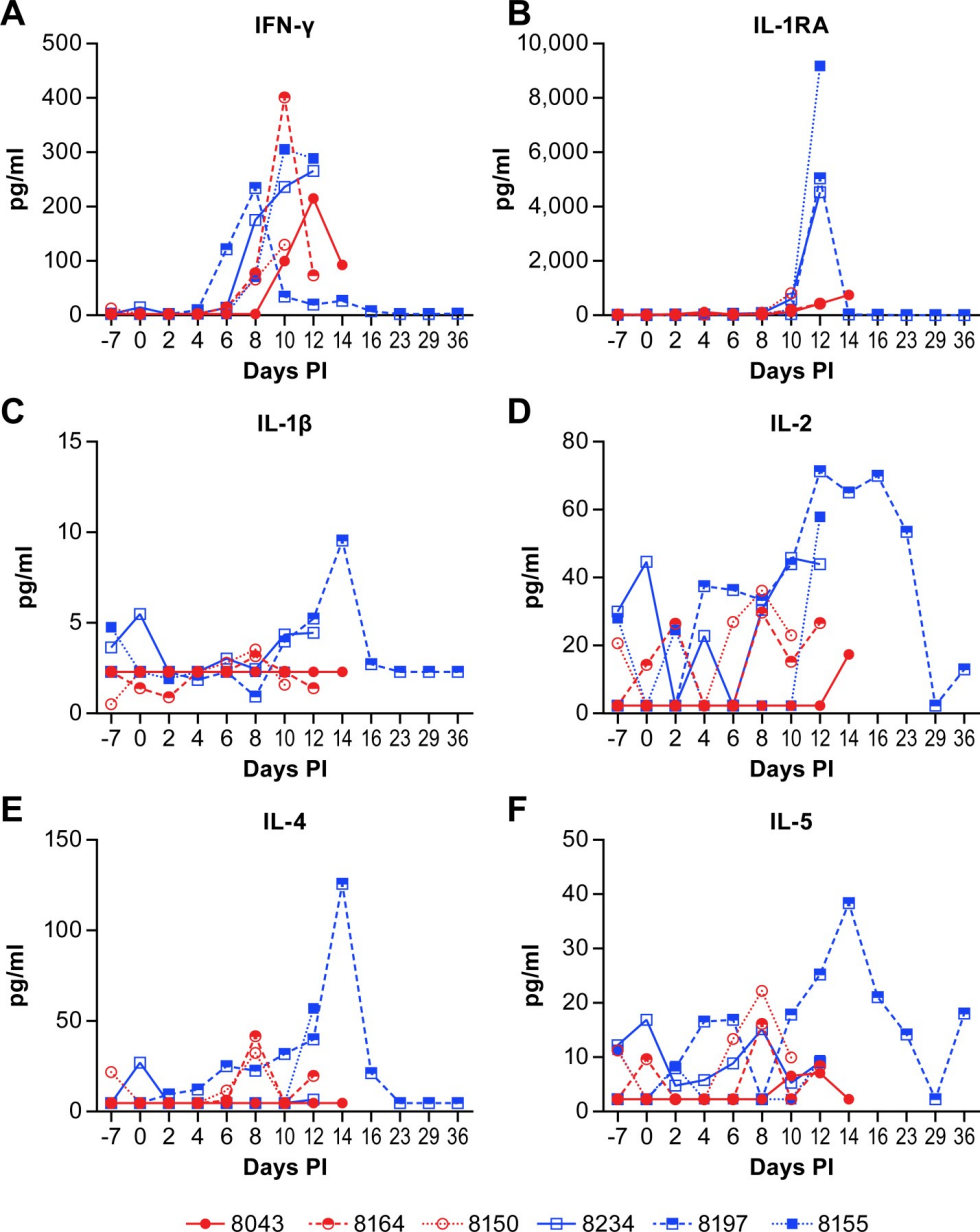
Plasma cytokine levels in animals infected with NiV. IFNγ (A), IL-1RA (B), IL-1β (C), IL-2 (D), IL-4 (E) and IL-5 (F) levels in individual animals over the course of disease following NiV infection. Animals in the low dose group are displayed as red circles and those in the high dose group as blue squares. Values represent the mean of triplicate technical replicates.

**Fig 3:**
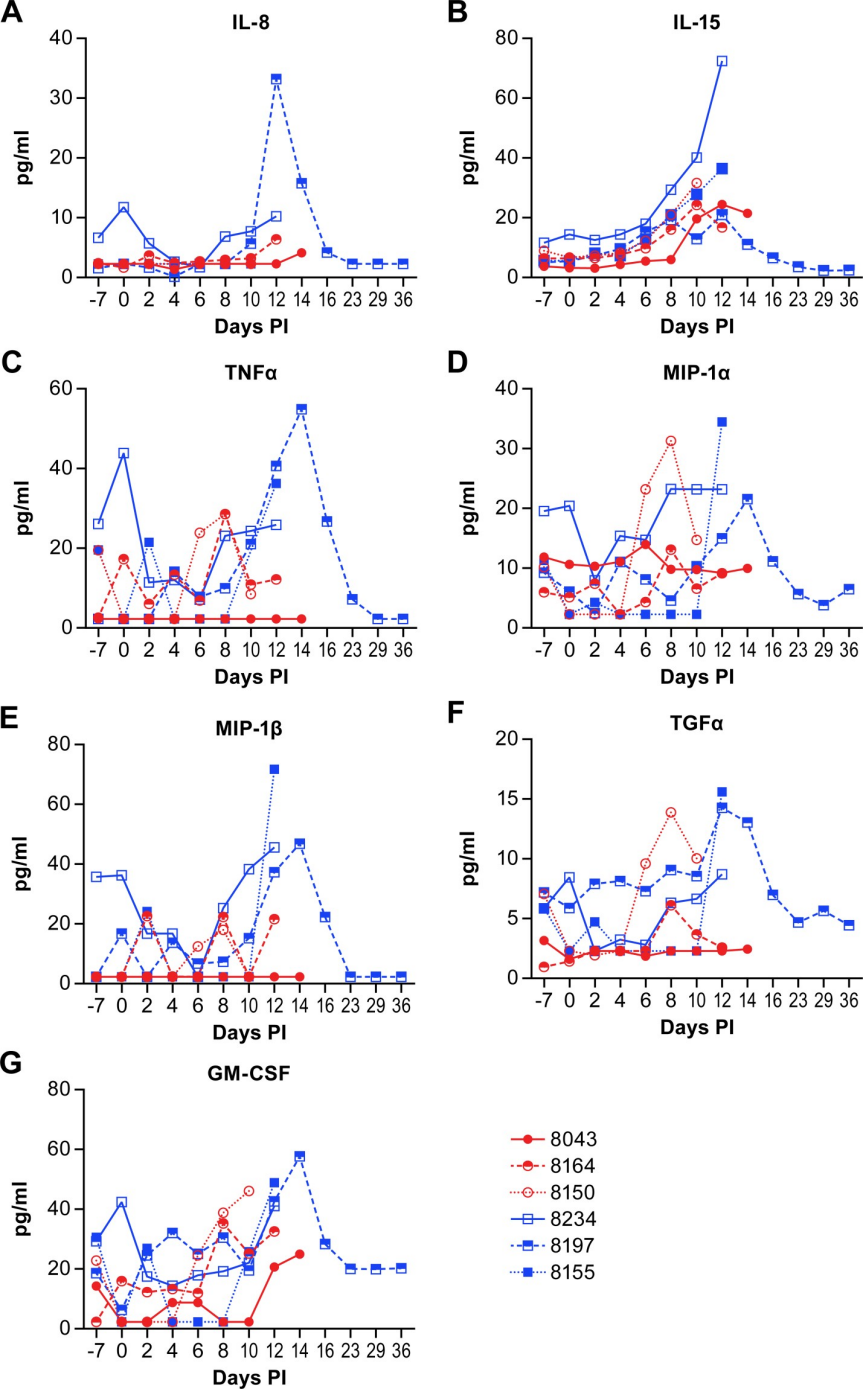
Plasma cytokine and chemokine levels in animals infected with NiV. IL-8 (A), IL-15 (B), TNFα (C), MIP-1α (CCL3) (D), MIP-1β (CCL4) (E), TGFα (F) and GM-CSF (G) levels in individual animals over the course of disease following NiV infection. Animals in the low dose group are displayed as red circles and those in the high dose group as blue squares. Values represent the mean of triplicate technical replicates.

Plasma cytokine levels were measured temporally over the course of disease using a bead-based multi-plex assay. The assay used in this study was a standard array that evaluates a broad range of cytokines and chemokines. There were few consistencies between the individual animals that could be used to characterize the disease process, with the exception of increased production of IFN-γ in all six animals between days 6 and 14 post-infection (Fig 2A). There were also some apparent dose specific responses with peak IFN-γ levels generally higher in high dose group animals (Fig 2A, blue squares). The IL-1RA levels were markedly higher in all three high dose group animals at 12 days post-infection while levels in the low dose group remained much lower (Fig 2B). Similarly, peak levels of IL-1β, IL-2, and MIP-1β were higher in all three high dose group animals than any of the low dose group animals (Figs 2C, 2D and 3E).

An opportunity from these studies was the ability to evaluate the disease process in an animal that survived NiV infection. This animal had marked increases in several cytokines and chemokines between days 10 and 16 post-infection that correlate with increases in IgG production and improved clinical health. These cytokines included IL-1β, IL-1RA, IL-2, IL-4, IL-5, IL-8, IL-15, TNFα, MIP-1α (CCL3), MIP-1β (CCL4), TGFα and GM-CSF (Figs 2 and 3). The increases in IL-2, IL-4, IL-5, GM-CSF and MIP-1β are all potentially associated with the transition from innate to adaptive immunity. The proinflammatory IL-8 and TNFα may be elevated in an effort to manage the systemic vasculitis that is common in this model of NiV infection [10, 11, 13, 14], although, in some circumstances, excessive TNFα may exacerbate vasculitis [19].

### Peripheral immune cell populations

There was little difference in the peripheral immune cell populations between the high and low dose challenge groups in this study. However, there were differences between individual animals, specifically the survivor, that may be critical for understanding viral inhibition of host immunity or specific immune characteristics that are important for survival of NiV infection.

**Fig 4:**
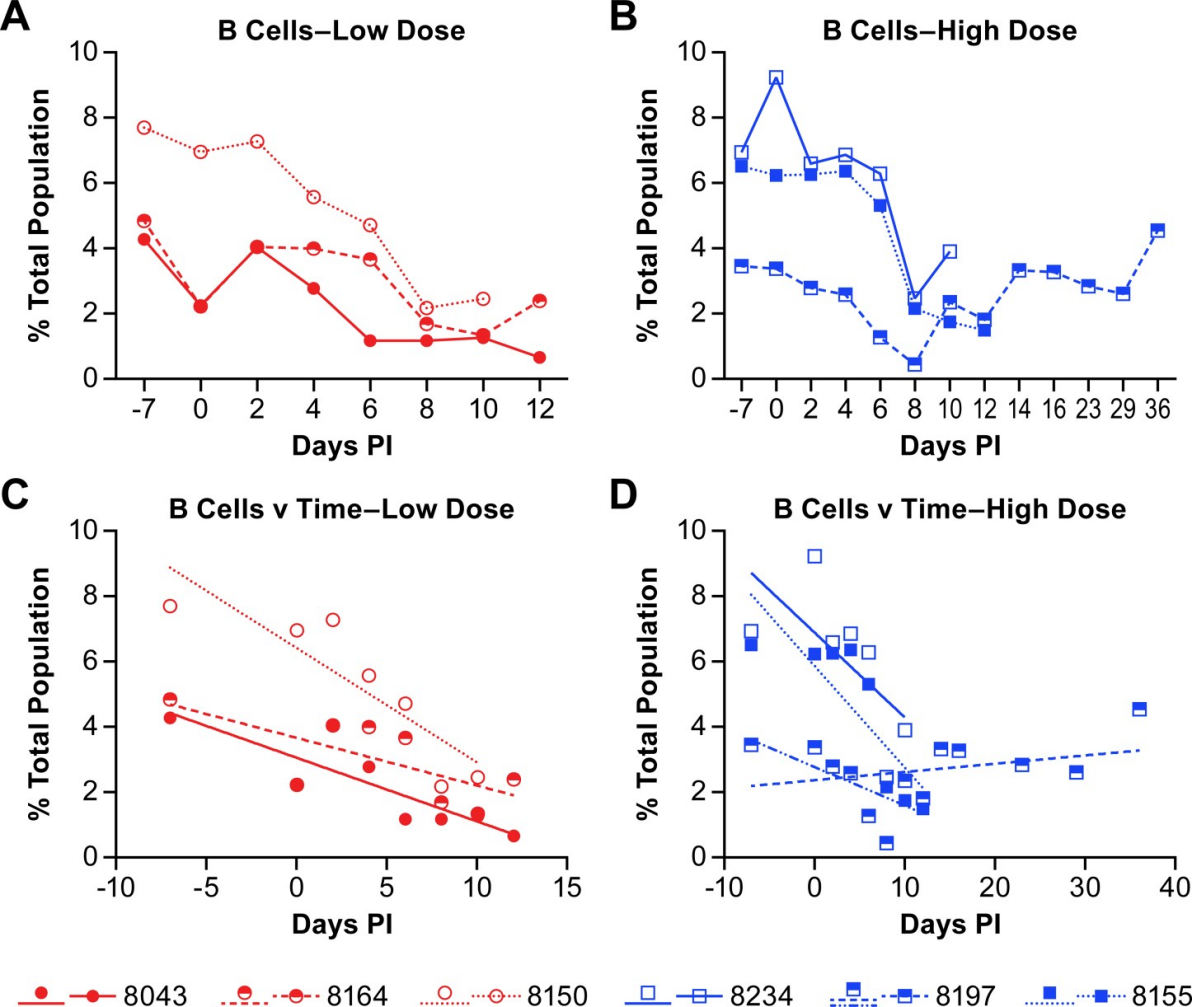
Changes in B cell populations in animals infected with NiV. The percent of B cells in the total population of analyzed cells is presented for individual animals in the low dose group (A and C) and the high dose group (B and D). Linear regression analyses (C and D) to determine the rate of change over the course of acute disease for all animals and convalescence for the animal that survived infection. The r-values for the linear regression analyses are provided in S1 Table. Linear regression analysis was performed on samples from all animals through day 12 (acute phase) with additional analysis of the surviving animal over the entire course of disease (blue dashed line).

In all six animals the B cell populations decreased over the course of the acute disease (defined as 0–12 dpi) with populations starting to recover in four of the six animals beginning at 10 dpi (Fig 4). There may be some correlation between the rate of B cell depletion and disease progression as the three animals with the shortest disease course (8150, 8234, 8155) also had the highest starting populations of B cells and the most rapid decline in population (Fig 4C and 4D; S1 Table).

**Fig 5:**
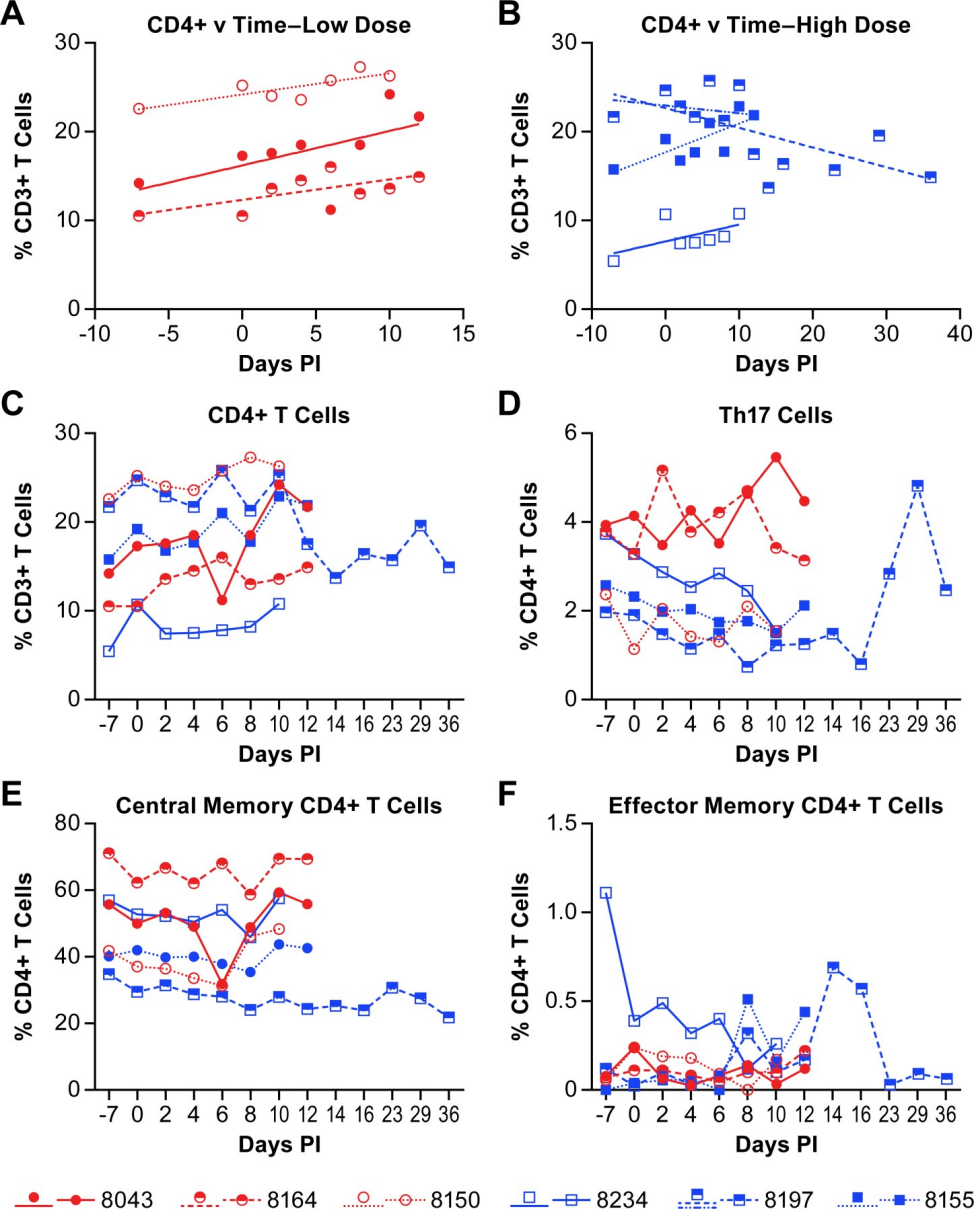
Changes in CD4+ T cell populations in animals infected with NiV. The percent of CD4+ T cells within the CD3+ cell population of individual animals is provided in panels A-C. Linear regression analysis of the low dose group (A) and high dose group (B) was performed to determine the rate of change of the CD4+ T cell population over the course of disease. The r-values for the linear regression analyses are provided in S1 Table. Linear regression analysis was performed on samples from all animals through day 12 (acute phase) with additional analysis of the surviving animal over the entire course of disease (blue dashed line). The population of Th17 cells (D), central memory CD4+ T cells (E) and effector memory CD4+ T cells (F) were determined within the total population of CD4+ cells. The cell surface markers for defining individual populations is provided in S3 Table.

The peripheral populations of CD4+, CD4+ Th17 and CD8+ T-cells were also quantified. In five of six animals the overall population of CD4+ T cells increased over the course of the disease while in the survivor (8197), the CD4+ T cell population decreased, even when evaluated over only the acute phase of disease (Fig 5A–5C, S1 Table). The population of Th17 cells was nominally decreased in some animals over the acute phase of disease, but unchanged in others (Fig 5D). The population of Th17 cells did increase in the convalescent animal with a peak at 29 days post-infection. However, the identification of Th17 cell populations did not include intracellular staining for IL-17 (see S1 Table) so additional cell populations may be included in these analyses. Further delineation of CD4+ populations into central and effector memory cells (see S1 Table) found that the central memory CD4+ T cells were least abundant in the survivor, but that populations were largely unchanged over the course of the study (Fig 5E). Baseline values of CD4+ central memory populations were consistent with previously published work [20]. Although the effector memory CD4+ T cells represented a very small percentage of the CD4+ T cells isolated from PBMCs, there was an increase in this population in the surviving animal on days 14 and 16 (Fig 5F).

**Fig 6:**
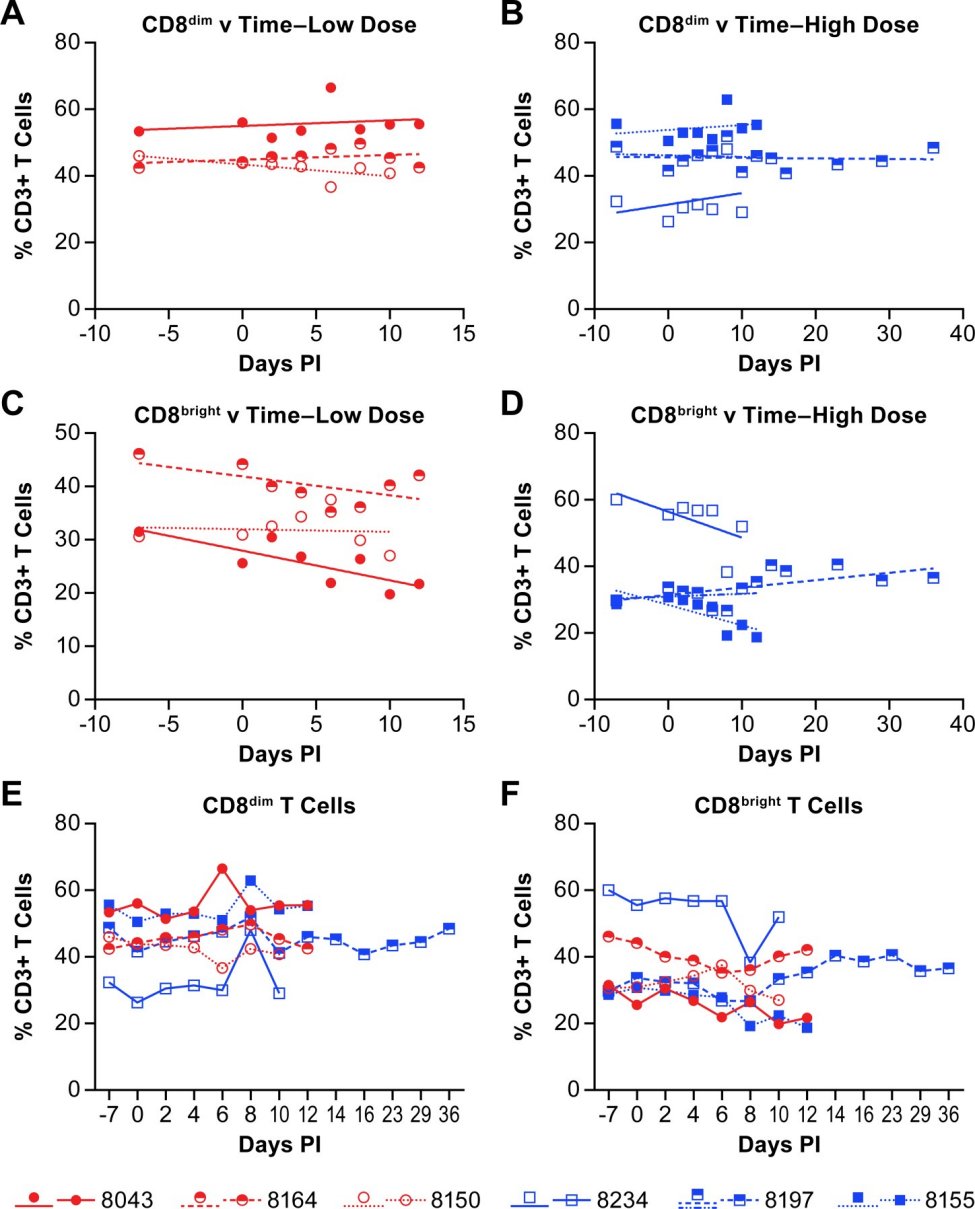
Changes in CD8+ T cell populations in animals infected with NiV. Linear regression analysis of the percent of CD8^dim^ (A, B) and CD8^bright^ (C, D) T cells within the CD3+ cell population of individual animals are shown for both the low (A, C) and high (B, D) dose groups over the course of disease. The r-values for the linear regression analyses are provided in S1 Table. Linear regression analysis was performed on samples from all animals through day 12 (acute phase) with additional analysis of the surviving animal over the entire course of disease (blue dashed line). Panels E and F show the populations of CD8^dim^ and CD8^bright^ T cells, respectively where red circles indicate the low dose group and blue squares indicate the high dose group.

Quantification of CD8+ T cell populations distinguished the previously established CD8^bright^ (CD8αβ) and CD8^dim^ (CD8αα) populations that are found in AGMs [21]. The CD8^bright^ populations serve a typical CD8+ functional role in adaptive immunity. The CD8^dim^ population appears to arise due to down-regulation of CD4 on T cells, but the cells retain some of their CD4 helper functions [20, 22]. There was a nominal increase in the CD8^dim^ T cell population in four of six NiV infected AGMs, while there was a slight decrease in the remaining two animals (Fig 6A, 6B and 6E; S1 Table). The population of CD8^bright^ T cells decreased in four of six animals, while in one of the animals the slope of the population change was approximately 0 (Fig 6C, 6D and 6F; S1 Table). The remaining animal, the survivor, had a nominal increase in the CD8^bright^ population over the acute phase of disease and the population increased further as the animal convalesced (Fig 6D and 6F; S1 Table).

**Fig 7:**
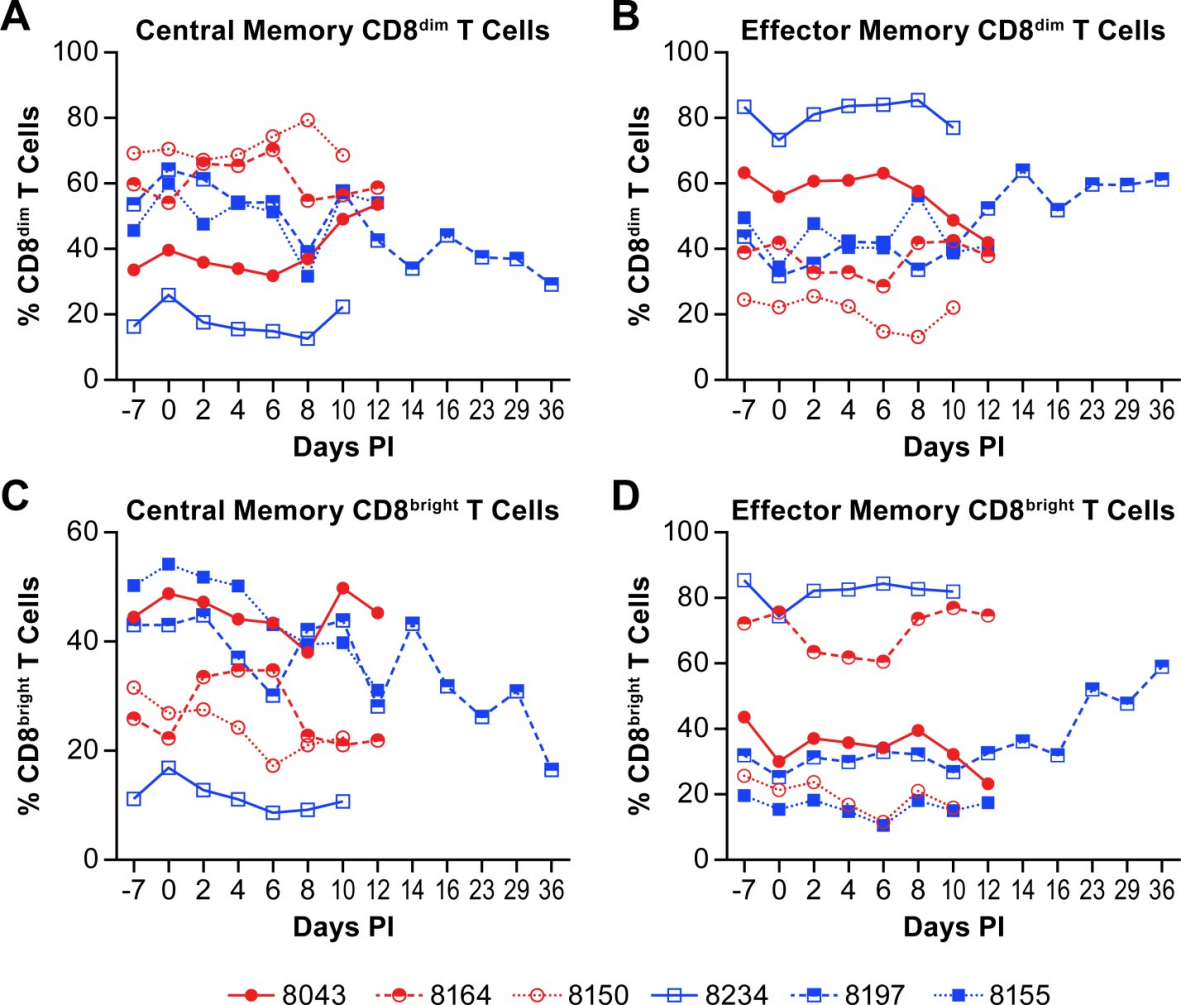
Changes in CD8+ memory T cell populations in animals infected with NiV. Plots show changes in the percent of CD8^dim^ central (A) and effector (B) memory populations in the total population of CD8^dim^ T cells and CD8^bright^ central (C) and effector (D) memory populations in the total population of CD8^bright^ T cells from individual animals infected with NiV. Red circles indicate the low dose group and blue squares indicate the high dose group. The cell surface markers for defining individual populations is provided in S3 Table.

To further delineate CD8+ T cell populations, CD8+ central and effector memory T cells were quantified over the course of disease. The populations of CD8^dim^ central memory cells either increased slightly or were essentially unchanged over the acute phase of disease in 5 of the 6 animals (Fig 7A). In the remaining animal, the survivor, the CD8^dim^ central memory population decreased over the acute phase of disease and continued to decrease during convalescence. The populations of effector memory cells were essentially the opposite with decreases in 5 of 6 animals and an expansion in the surviving animal (Fig 7B).

The populations of CD8^bright^ central memory cells decreased over the course of the disease in all animals, with no change in the rate of decrease in the survivor during convalescence (Fig 7C). The change in populations of effector memory CD8^bright^ T cells was minimally variable over the acute phase of disease with all but one animal (8043) having similar levels of this cell population at baseline and termination (Fig 7D). The population of CD8^bright^ effector T cells increased markedly during convalescence of the survivor as one might expect for clearance of residual infected cells.

**Fig 8:**
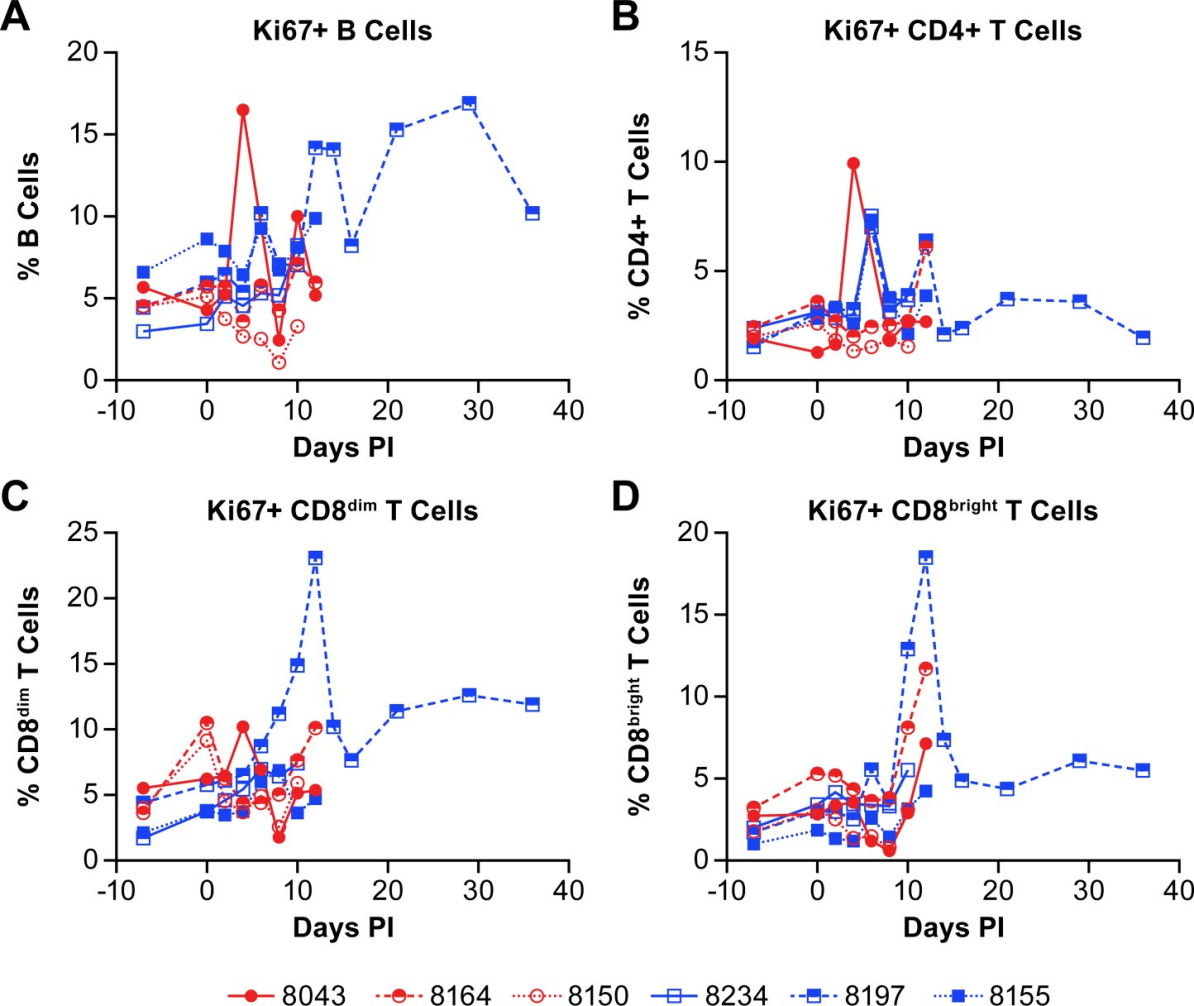
B and T cell proliferation in animals infected with NiV. The percent of proliferating (Ki67+) (A) B cells, (B) CD4+ T cells, (C) CD8^dim^ T cells and (D) CD8^bright^ T cells are shown as the total population of the analyzed cells from individual animals. Red circles indicate the low dose group and blue squares indicate the high dose group.

Evaluation of the populations of proliferating B and T cells based on expression of the Ki67 marker found that, in agreement with the increase in population of B cells in the convalescent animal, the percentage of Ki67+ B cells increased markedly beginning on day 12 post-infection (Fig 8A). Furthermore, the percentage of Ki67+ CD4+ T cells changed little over the course of acute disease, despite some fluctuation in individual animals (Fig 8B). There was a slight but sustained increase in proliferating CD4+ T cells in the surviving animal early in convalescence. The proliferation of CD8+ T cells, however, showed a marked increase in the percentage of Ki67+ CD8+ T cells in the survivor late in the acute phase of disease that was sustained during convalescence (Fig 8C and 8D). The population of Ki67+ CD8^bright^ T cells was also increasing in two of the animals that succumbed to disease (Fig 8D).

**Fig 9:**
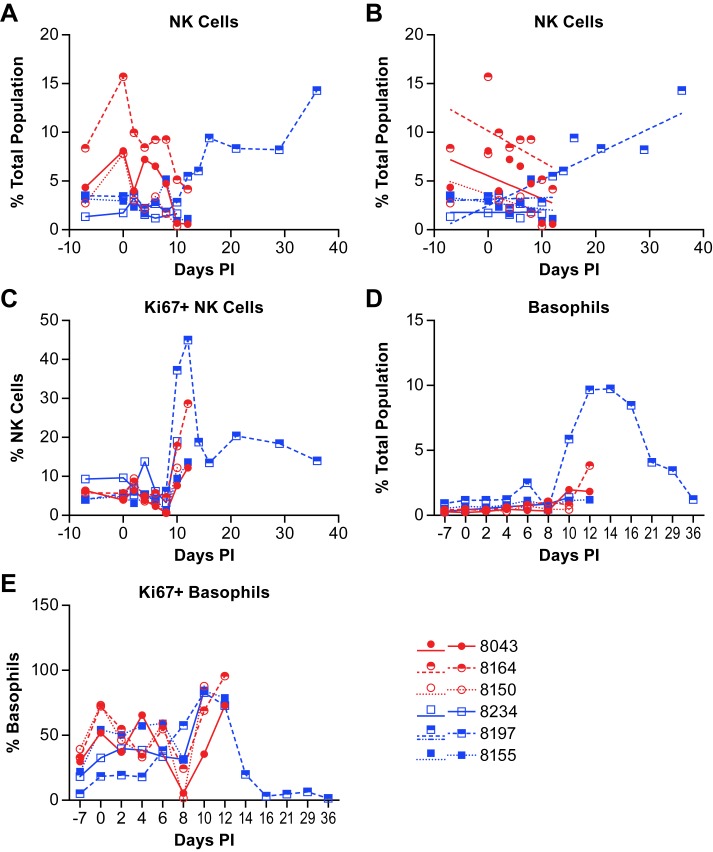
Changes in antigen presenting cell population in animals infected with NiV. The percent of monocytes (A), myeloid dendritic cells (mDC) (B) and plasmacytoid dendritic cells (pDC) (C) are shown as a function of the total population of analyzed cells from individual animals. Red circles indicate the low dose group and blue squares indicate the high dose group. The cell surface markers for defining individual populations is provided in S3 Table.

Additional peripheral immune cell populations evaluated included NK cells, monocytes, myeloid dendritic cells (mDC) and plasmacytoid dendritic cells (pDCs). Monocytes represented 2–6% of the total leukocyte population in the blood at baseline. Linear regression analysis of this population suggested that the population trended downward over the acute phase of disease in all of the animals except the survivor, which trended slightly upward, but these analyses were complicated by marked day-to-day variability of the analyzed populations in some animals (Fig 9A; S1 Table). The data for the survivor are somewhat skewed as this population trended downward until 8 dpi and was elevated on dpi 10–14 before dropping to baseline levels (Fig 9A; S1 Table). Myeloid and pDCs made up 1–3 and less than 0.5 percent of the total non-erythrocyte population, respectively, at baseline (Fig 9B and 9C; S1 Table). There was little consistent change in the mDC and pDC populations over the acute phase of disease. The mDC population was largely unremarkable. However, three animals (8164, 8197, 8155) had similar increases in their pDC populations on days 10–12, with the survivor (8197) maintaining the increased level through 16 dpi before returning to baseline levels.

**Fig 10:**
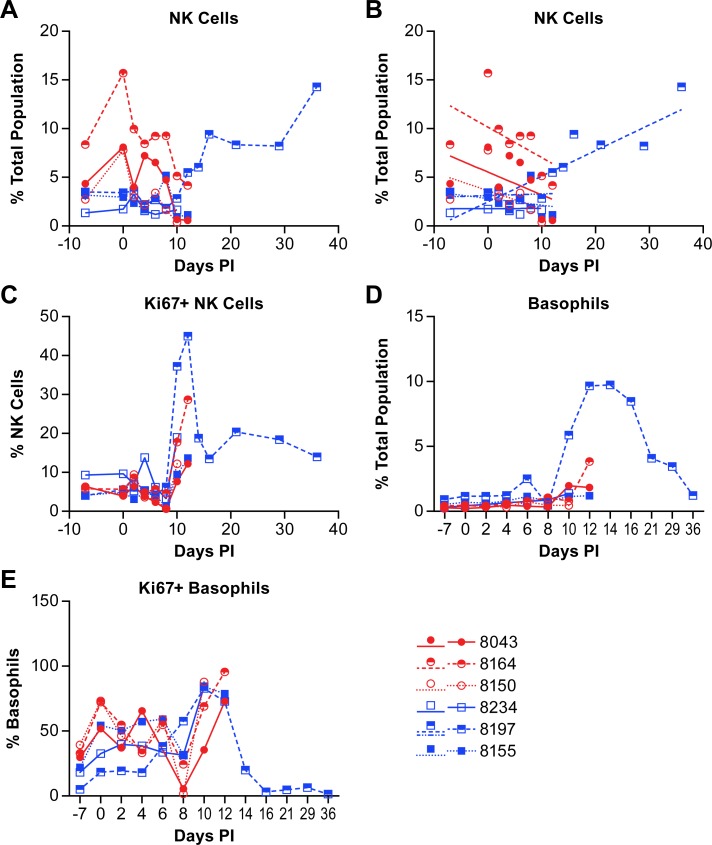
Changes in the populations of NK cells and basophils in animals infected with NiV. Data shows the changes in NK cell populations (A) within the total population of analyzed cells from animals infected with NiV over the course of disease and linear regression analysis (B) of these same populations. Linear regression analysis was performed on samples from all animals through day 12 (acute phase) with additional analysis of the surviving animal over the entire course of disease (blue dashed line). The level of Ki67+ (proliferating) NK cells (C), basophils (D) and Ki67+ basophils (E) were also evaluated. Red circles indicate the low dose group and blue squares indicate the high dose group. The cell surface markers for defining individual populations is provided in S3 Table.

NK cell populations varied between individual animals over the course of acute disease. However, linear regression analysis shows that the NK cell population decreased in all three animals in the low dose group over the course of acute disease while two of three animals in the high dose group were essentially unchanged and the third decreased nominally (Fig 10A and 10B; S1 Table). In the surviving animal, the NK cell population was essentially unchanged through day 10 post-infection (Fig 10A and 10B), but then increased on day 12 and remained elevated through study termination. Evaluation of NK cell proliferation through expression of Ki67 found an increase in the percentage of proliferating NK cells in most animals on days 10–12 post infection (Fig 10C). The proliferating NK cell population in the surviving animal peaked at day 12 with over 40% of the cells Ki67+. The percentage of Ki67+ NK cells decreased again by day 14 but remained well above baseline levels through study termination.

Evaluation of the basophil population (Fig 10D; S1 Table) found that there were no marked changes over the course of the acute disease, but there were elevations in several animals in the late phase of disease as the animals became terminal (Fig 10D). The surviving animal had a large increase in the basophil population beginning at day 10 post-infection with a peak at day 14 and gradual decline as the animal convalesced. Using Ki67 as a proliferation marker, most basophils on days 10–14 appeared to be proliferating (Fig 10E).

**Fig 11:**
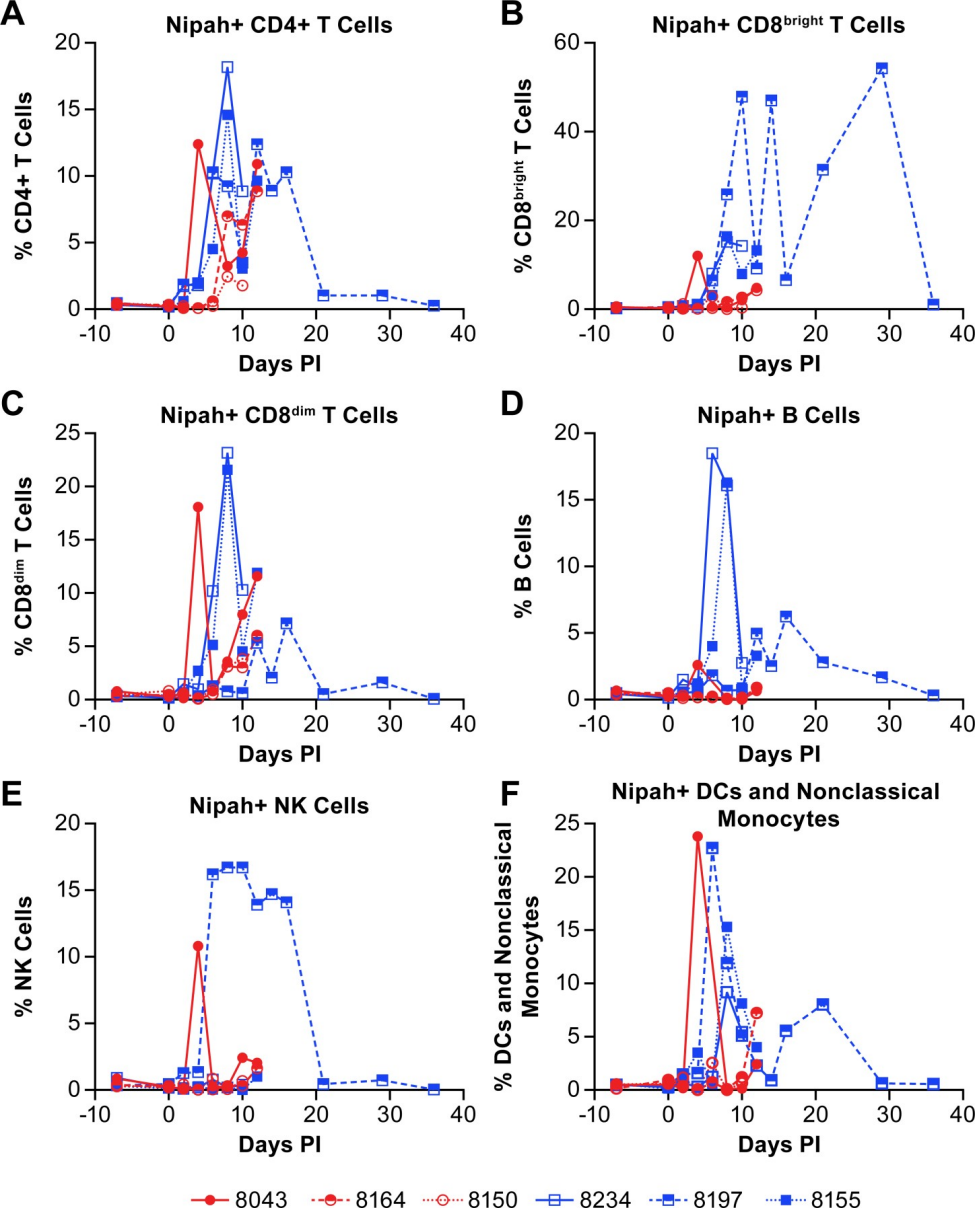
Determination of cell populations containing NiV antigen. The presence of NiV was determined in individual cell populations by flow cytometry. Antigen was identified in CD4+ T cells (A), CD8^bright^ T cells (B), CD8^dim^ T cells (C), B cells (D), NK cells (E) and a combined population of DCs and non-classical monocytes (F). Red circles indicate the low dose group and blue squares indicate the high dose group. The cell surface markers for defining individual populations is provided in S3 Table.

As a component of the analysis of peripheral immune cell populations, the presence of NiV antigen was determined in individual cell populations using a NiV-M glycoprotein specific polyclonal antibody. While not consistent in all animals, and dependent upon the point in the disease course, viral antigen was detected in CD4+ T cells, CD8^bright^ T cells, CD8^dim^ T cells, B cells, NK cells and DCs/nonclassical monocytes (Fig 11). Interestingly, the surviving animal had the most robust and persistent increase in NiV antigen positive NK cells (Fig 11E). While the presence of viral antigen is intriguing, this does not mean these cell populations are infected and that virus is replicating. However, the persistent presence of NiV in some peripheral cell populations, and NK cells in particular, is suggestive of viral replication. Specific analyses to determine if NiV was replicating in these cell types are a component of on-going studies.

## Discussion

In this project our objective is to develop a model of NiV infection that is similar to both the disease course and survival rate that is seen in humans. Previous work using IT or small particle aerosol infections led to rapidly progressing pulmonary disease with 100% lethality [10, 11]. The IT and small-particle inoculation routes provide either a bolus of virus or drive the virus deep into the lungs, neither of which is likely to represent probable human exposure routes. In the initial outbreak in Malaysia and Singapore, humans were likely infected through contact with or inhalation of respiratory excreta from pigs. In Bangladesh and India, the primary routes of infection are through consumption of contaminated date palm sap or direct human-to-human contact. There is no evidence to support alimentary infection and it is unlikely that the virus would remain infectious in the low pH environment of the stomach. Subsequently, our hypothesis is that the virus either enters through open wounds in the mouth or it is distributed into the lungs or sinuses through either accidental inhalation or coughing [23].

In the current study we used aerosol exposure with an intermediate-size particle (~7 um) to focus virus deposition into the upper respiratory tract and sinuses [24]. The analyses described here focused on the impact of NiV infection on serum cytokine expression and changes in peripheral immune cell populations. Critical aspects of these findings demonstrate that there were no specific correlates of the acute disease that were uniform among the animals tested in this study, with the exception of increased levels of IFNγ toward the end of the acute disease phase. However, the lack of a systemic inflammatory cytokine response highlights the potential importance of local inflammation in disease development and control and belies our expectation that extensive vasculitis would lead to a systemic inflammatory response. Furthermore, the limited adaptive immune response prior to day 12 in all but the surviving animal suggests that rapidly developing adaptive immunity may be important to surviving NiV infection in this model. However, the change in CD4+ T cell population is in contrast to findings from studies using a porcine model of NiV infection [25] where a decrease in the CD4+ CD8- populations was associated with a poor outcome. Here, we found that the disease course was delayed relative to previous work and that the pulmonary disease was less severe, despite 5 of 6 animals succumbing to the disease [14]. The animal that survived the infection had an immune response that was different from the other five animals in many respects. Specifically, these differences were in the kinetics of the CD4+ and CD8+ T cell responses over the acute phase of disease, and an earlier increase in its B cell population after an initial decrease. The differentially regulated T cell response may have allowed the surviving animal to develop an earlier transition from innate to adaptive immunity allowing a more rapid expression of virus specific IgM and induction of protective immunity. While these data are for only a single animal, they provide useful insights for areas of additional research into mechanisms regulating the development of adaptive immunity in this animal model. Similar to the work reported here, previous studies in the AGM and ferret models of NiV infection don’t generally indicate a marked increase in virus specific antibody titers until around days 7–10 post infection where virus neutralization was evident [13, 26, 27]. In these studies, most of the surviving animals were either in a therapeutic treatment group or had been infected with an attenuated virus which allowed for an extended disease course.

The kinetics of the CD4+ T cell response in the surviving animal is interesting as this population decreased over the acute phase of the disease and continued to drop during convalescence. The CD4+ T cell population in all other animals increased over the acute phase of disease which one might expect if developing an adaptive immune response. The surviving animal had an increase in IL-2 beginning around day 4 that may be correlated with a decrease in CD4+ T cells as this has been shown *in vitro* [28, 29]. These data suggest an early Th1 response in the acute phase of disease in this animal, similar to what we previously reported [10], followed by a Th2 response during convalescence. This hypothesis is supported by the production of Th1-associated IgG1, although testing for the Th2-associated IgG4 was not performed. In addition, in the AGM model, CD4 downregulation has been shown to correlate with an increase in CD8^dim^ T cells in the context of simian immunodeficiency virus (SIV) which retain CD4 functionality, but also develop cytotoxic activity of classical CD8+ T cells [22, 30]. In this study, the CD8^dim^ population was largely unchanged over the course of disease in all of the animals, but this may be due to the acute nature of the disease, whereas SIV is a chronic infection. Decreases in lymphocyte populations may also be associated with homing to lymphoid tissue, but this question was not specifically addressed in this study. The surviving animal also provided insights to T cell changes during convalescence including a nominal increase in CD8^dim^ effector memory cells.

Interestingly, a recent study examining B and T cell populations in two survivors of NiV infection in India [31] identified activation of both CD4+ and CD8+ T cells and proliferation (Ki67+) of CD8+ T cells in a response that is similar to that seen in the surviving animal in the study reported here. While the survivor study examined a very limited population (n = 2) and the study reported here identified only a single NHP survivor, the correlations between the human and NHP B and T cell responses is compelling and potentially identifies key correlates of protective immunity.

Evaluation of the NK cell population found considerable variability between animals across the acute phase of disease. Proliferation of NK cells, based on expression of Ki67, began around 10 days post-infection with the peak percentage of Ki67+ NK cells on either 10- or 12-days post-infection, depending on the terminal day of the disease course. In the survivor, the peak NK population among the total cells analyzed was at the end of the study on day 36, however the peak Ki67+ population was on day 12 suggesting that NK cells were no longer proliferating, but that populations of other cells were declining. Interestingly, approximately 15% of the NK cells identified in the survivor were NiV antigen positive from 6–16 days post-infection (Fig 11E). While there is no direct evidence, other than the presence of viral antigen, supporting NiV infection of NK cells in this study, previous work with a porcine model has shown that NiV can infect and replicate in porcine NK cells, albeit at low titers [25]. If this report is accurate, it could be surmised that NiV is infecting NK cells but not until the late acute to convalescent phase of disease. This finding may suggest that NK cell activation is required for the cells to become permissive to NiV infection. Levels of IL-15, a cytokine that stimulates NK cell proliferation [32], were elevated above baseline 6–12 days post-infection but were lower than in other animals during the same time frame (Fig 3B). Stimulation of NK cells induces the release of TNFα and IFNγ from NK cells with IFNγ release leading to the upregulation of MHC II on numerous cell types for activation of CD4+ T cells. While the level of IFNγ peaked at day 8 in the survivor (Fig 2A), TNFα was elevated late in the acute phase of disease (days 10–14) and returned to baseline by the end of the study (Fig 3C), similar to the percentage of NiV antigen positive NK cells. These data suggest activation, proliferation and functional activity of NK cells during both the acute and convalescent phase of NiV infection in the surviving animal. A similar response profile during the acute phase was not evident in animals that succumbed to the disease.

In addition to increased NK cells during the late acute and convalescent phase of disease, basophil populations in the survivor increased dramatically beginning 10 days post-infection, peaked at day 14 and gradually returned to near baseline levels at study termination (Fig 10D). Only one other animal had moderately elevated basophil populations that peaked at its terminal day. Evaluation of basophil proliferation using the Ki67 marker varied among animals during the acute phase of disease. All of the animals reached at least 70% of the population positive for Ki67 on days 10–12 post-infection (Fig 10E). The levels of GM-CSF, which stimulates production of granulocytes from stem cells and can stimulate maturation of dendric cells and differentiation of granulocytes [33], was elevated above baseline in several of the infected animals and peaked in the survivor at day 14 post-infection (Fig 3G). IL-4, which is produced by basophils was elevated above baseline beginning around 6 days post-infection in the surviving animal, and peaked at day 14, similar to the peak population of basophils in this animal (Figs 2E and 10D). These data demonstrate that the basophil population was stimulated to increase during NiV infection in the surviving animal, possibly by elevated production of GM-CSF, and that these cells were potentially a source of IL-4 in the plasma.

Previous work by Mathieu *et al*. demonstrated that NiV binds human leukocytes and suggested that these cells were a means of virus dissemination [34]. Additionally, Stachowiak and Weingartl found that NiV was able to productively infect porcine CD6+CD8+ T cells and NK cells [25]. Here we identified NiV antigen in leukocyte populations including CD4+ and CD8^dim^ T cells of all of the animals analyzed, as well as in CD8^bright^ T cells, B cells, NK cells and DC/non-classical monocyte populations. While these data do not clearly demonstrate infection *in vivo*, combined with the work of Mathieu *et al*., it is suggestive that NiV is able to either bind to or infect multiple human leukocyte populations. Previous work with measles virus (MV), a related paramyxovirus, has shown that MV can infect human B cells *in vitro* [35] and B and T cells *in vivo* in a macaque model [36] and has been proposed as a potential means of immune suppression. While the route of MV infection of lymphocytes is believed to be through binding with its CD150 receptor, it is possible that NiV has developed a similar means of immune suppression by stimulating lymphocyte depletion. Ephrin B2 has been shown to be expressed in *in vitro* differentiated human Th1, Th2 and Th17 cells [37]. Ephrin B2 is also found on thymocytes in mice [38] and CD3+ T cells isolated from humans [39]. However, it is not clear if ephrin B2 or ephrin B3 are expressed on peripheral human or AGM CD4+ or CD8+ T cells and if so, at what levels. This question will be addressed in future studies. In addition, the presence of NiV in NK cells or lymphocytes well after the disease has ended may indicate a potential source of viral persistence or chronic infection as there have been reported cases of delayed or recurrent encephalitis in some NiV patients [40]. Histopathological assessment of animals included in this study demonstrated neurological involvement, although evidence of virus infection in the brain was limited [14].

This work provides the first comprehensive evaluation of the peripheral immune response in the AGM model of NiV infection. The results shown here clearly demonstrate some commonalities in the response to acute lethal disease and also suggests potential immune correlates to survival of NiV infection. These studies, combined with previous work, suggest that immunopathogenesis associated with NiV infection is likely to be focal as there no clear evidence of a large systemic response. These studies also suggest the potential importance of rapid development of virus-specific adaptive immunity for survival of NiV infection. While these studies do not provide definitive examples of NiV-driven immune modulation, they do indicate that a more focused approach to understanding the regulation of the immune response to NiV infection and the regulation of host immunity by NiV is critical to managing this disease.

## Methods

### Animals

Wild-caught Caribbean origin AGM were purchased from PrimGen (Hines, IL). Animals were identified for inclusion based on similarity of size with one male and two females included in each group. Group sizes (n = 3) were limited due to clinical imaging requirements associated with this study where each imaging session was approximately 2 hours per animal [14]. Animals were group housed prior to being assigned to the study and were singly housed during the course of the study. At all times animals were provided with appropriate enrichment including, but not limited to, polished steel mirrors and durable toys. Animals were anesthetized in accordance with BSL-4 standard protocols prior to all procedures including inoculation, imaging and collection of blood to minimize stress to the animals. Animals were observed following anesthesia to ensure complete recovery.

### Ethics statement

Work with non-human primates was conducted in accordance with an Animal Study Protocol approved by the NIAID Division of Clinical Research Animal Care and Use Committee following recommendations in the Guide for the Care and Use of Laboratory Animals. This institution also accepts as mandatory the Public Health Service policy on Humane Care and Use of Laboratory Animals. All animal work at NIAID was performed in a facility accredited by the Association for the Assessment and Accreditation of Laboratory Animal Care International (AAALACI). All work with non-human primates was done in accordance with the recommendations of the Weatherall Report.

### Virus and cell culture

The Malaysian strain of NiV that was used in this study was isolated from a fatal human case in 1998 [41]. This virus stock was obtained from a collection housed at the US Army Medical Research Institute for Infectious Diseases and has a documented passage history that includes three passages in Vero E6 cells, one passage in Vero cells and two additional passages in Vero E6 cells. The stock virus sequence information is available through GenBank (Accession #KY425646.1) and is consistent with the previously published sequence for this virus (Accession #AF212302). Use of this virus stock is exempt from IRB approval requirements.

VeroE6 cells (BEI #NR596) were maintained at 37°C/5% CO_2_. in α-MEM w/GlutaMAX and containing 10% fetal bovine serum (FBS). All work with viable NiV was performed in the BSL-4 facility at the NIAID Integrated Research Facility in Frederick, MD.

### Viral exposure

Animals were exposed to a NiV aerosol with a mean particle size of approximately 6.76 um. The low dose group received an average presented dose of 63.68 PFU and the high dose group received an average presented dose of 701.50 PFU. The specific aerosol exposure protocol is described elsewhere in a companion paper to this study [14].

### ELISA

IgM and IgG assays were developed in-house for use in determination of virus specific antibody titers. The viral antigen used in these assays was a cell extract generated from NiV-infected Vero E6 cells lysed with radioimmunoprecipitation (RIPA) buffer (Cell Signaling), irradiated with a 5 MRad total dose to inactivate the virus and sonicated to dissociate the lysate.

The IgM, IgG_1_ and IgG_2_ assays were antibody-capture ELISAs in which 96-well plates were coated with 0.2 μg/well anti-monkey IgM (mu) antibody (KPL #071-11-031), mouse anti-human IgG_1_ Fc (LSBio # LS-C351375) or mouse anti-human IgG_2_ (ThermoFisher #MA5-16715) diluted in PBS in a final volume of 100 μl. The plates were stored at 4°C overnight and not more than 2 weeks prior to use. The plates were washed 6 times with PBST (PBS containing 0.1% v/v Tween-20). Heat inactivated (56°C for 1 hour) test serum was diluted as appropriate in Serum Dilution Buffer (Blocking Buffer (PBST containing 3% normal chicken serum (Abcam) and 2% skim milk powder (ThermoFisher)) containing heat inactivated normal monkey serum (locally sourced AGM serum) and added to individual wells in a volume of 40 μl followed by overnight incubation at 4°C. The test serum was evaluated using a two-fold serial dilution in triplicate assays. The plates were washed 6 times with PBST and the NiV cell extract diluted in blocking solution 3:1 was added in a volume of 50 μl (IgM) or 100 μl (IgG_1_ and IgG_2_) per well. The plates were incubated at 37°C for 1h and then washed 6 times with PBST. A NiV glycoprotein (GP) specific rabbit antiserum (locally generated) was diluted 1:4000 in blocking solution and added to individual well in a volume of 100 μl and the plates incubated for 1 h at 37°C. The plates were washed 6 times with PBST and 100 μl HRP conjugated goat anti-rabbit antibody (Sigma #6154) diluted 1:10,000 in blocking buffer was added to each well. The plates were incubated for 1 h at 37°C and then washed 6 times with PBST prior to adding 100 μl of TMB substrate (ThermoFisher) and incubating at room temperature for 10–30 min before stopping the reaction with 100 μl Stop solution (ThermoFisher). Absorbance was read at 450 nm.

The IgG assays were direct antigen ELISAs where the plates were coated with NiV-infected cell extract diluted in coating buffer (BioLegend) at a concentration of 0.05 μg/well in a volume of 50 μl/well. The coated plates were stored at 4°C overnight and not more than 2 weeks prior to use. The plates were washed with 6 times with PBST and blocking solution added to each well prior to incubating at 37°C for 2 h. The plates were washed 6 times with PBST and heat inactivated test serum diluted in Serum Dilution buffer was added to the wells in a two-fold serial dilution, in triplicate wells in a volume of 40 μl/well. The plates were incubated at 4°C overnight and then the plates were washed 6 times with PBST. An HRP-conjugated rabbit anti-monkey secondary antibody (Sigma, #A2054) diluted in blocking buffer was added to each well in a volume of 100 μl and incubated at 37°C for 1 hour. The plates were washed 6 times with PBST and TMB substrate added for 10 minutes at room temperature before stopping the reaction with Stop solution. The absorbance was read at 450 nm.

### Neutralization assays

Neutralizing antibody titers were determined using a TCID_50_-based assay as previously described [42]. Briefly, test sera were serially diluted two-fold and incubated with an equal volume of virus (500 pfu/well) for 1 hour at 37°C. The virus-serum mixture was then added in triplicate to Vero E6 cells seeded a day prior at 4x10^5^ cells per well in a 96-well plate and incubated for 1 hour at 37°C to allow virus attachment and entry. Cell culture medium was added to a final volume of 200 μl and the cells incubated for 5 days at 37°C and 5% CO_2_. Following the incubation, the cell culture supernatant was removed, the cells washed with PBS and the cells fixed and stained with neutral buffered formalin (NBF) containing 0.25% crystal violet. The neutralization titer was determined as the endpoint where at least 50% of an individual well was clear.

### Cytokine response

The cytokine response in plasma was evaluated using a 23-plex bead based NHP cytokine panel (Millipore) on a FlexMap analysis system (Luminex) following the manufacturer’s instructions. All samples were run in duplicate wells. Data were analyzed and graphed using Excel and Prism 7.0 (GraphPad).

### Peripheral immune cell populations

Peripheral immune cell populations were evaluated by staining whole blood with a panel of antibodies designed to differentiate different immune cell populations. The red blood cells were lysed by resuspending pelleted cells with FACSLyse (BD Biosciences) and incubating the cells at room temperature in the dark for 10 min. The unlysed cells were pelleted by centrifugation and washed once with PBS-2 (PBS containing 2% FBS). The cells were resuspended in Cytofix/Cytoperm (BD Biosciences) and incubated for 30 min in the dark at room temperature. The cells were pelleted, resuspended in PermWash (BD Biosciences) and re-pelleted. Perm wash was added and the cells were pelleted. The cells were resuspended in the intracellular antibody cocktail and incubated at 4°C overnight in the dark. The next day, PermWash was added (2x previous volume) and the cells pelleted. The cells were resuspended in PermWash vortexed and were stored at 4°C until acquisition. The panel with specific antibody clone and fluorophore information is provided in S2 Table and the markers used to define individual cell populations is provided in S3 Table. Data were collected on an LSR Fortessa (BD Biosciences) and analyzed with FlowJo using FMOs to gate the negative controls for each antibody. The gating strategies used for these analyses are provided in S1 and S2 Figs. Final data were plotted using Prism (GraphPad).

### Data analysis and statistics

Data were analyzed using Excel (Microsoft) and or Prism (GraphPad). Linear regression analyses were performed using Prism 7.0. Statistical analysis was not performed in these studies given the limited statistical strength that would be provided by the small (n = 3) group sizes of outbred animals. Where mean values are provided, these are the mean of replicate samples within an assay and not within groups.

## Supporting information

S1 Fig Gating strategy for lymphocytes. The gating strategy used for differentiating lymphocyte populations was the same for all animals and all timepoints. Testing for the presence of NiV antigen was in addition to determinations for the populations defined here.
(TIF)

S2 Fig Gating strategy for monocytes and dendritic cells. The gating strategy used for differentiating monocyte and dendritic cell populations was the same for all animals and all timepoints. Testing for the presence of NiV antigen was in addition to determinations for the populations defined here.
(TIF)

S1 Table Calculated slopes for linear regression analyses. Linear regression analysis was performed on the specific cell populations of all animals through the acute phase of disease and additionally on the surviving animal for the complete course of disease.
(DOCX)

S2 Table Flow cytometry panel. Target cell surface markers, antibody clones and fluorphores used for the differentiation of peripheral immune cell populations.
(DOCX)

S3 Table Cell population phenotypes. Cell surface markers used to define individual cell populations in the performed analyses.
(DOCX)
