# Supplementary figures and images for: Peripheral immune response in the African green monkey model following Nipah-Malaysia virus exposure by intermediate-size particle aerosol
Source: PLoS Negl Trop Dis. 2019 Jun 5;13(6):e0007454. doi: 10.1371/journal.pntd.0007454 (PMC6576798; doi:10.1371/journal.pntd.0007454)

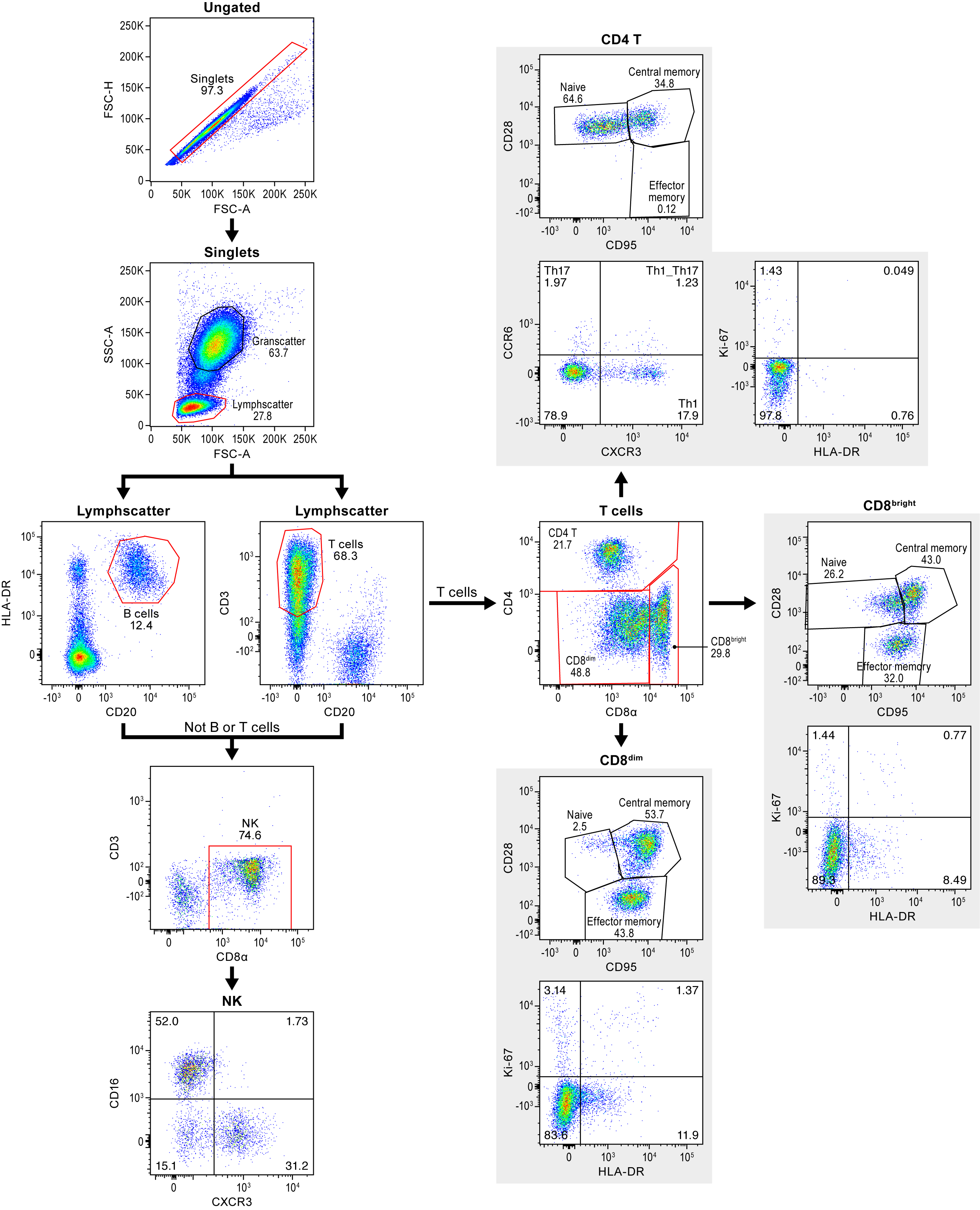

Supplement: S1 Fig — The gating strategy used for differentiating lymphocyte populations was the same for all animals and all timepoints. Testing for the presence of NiV antigen was in addition to determinations for the populations defined here. (TIF) [file pntd.0007454.s001.tif]

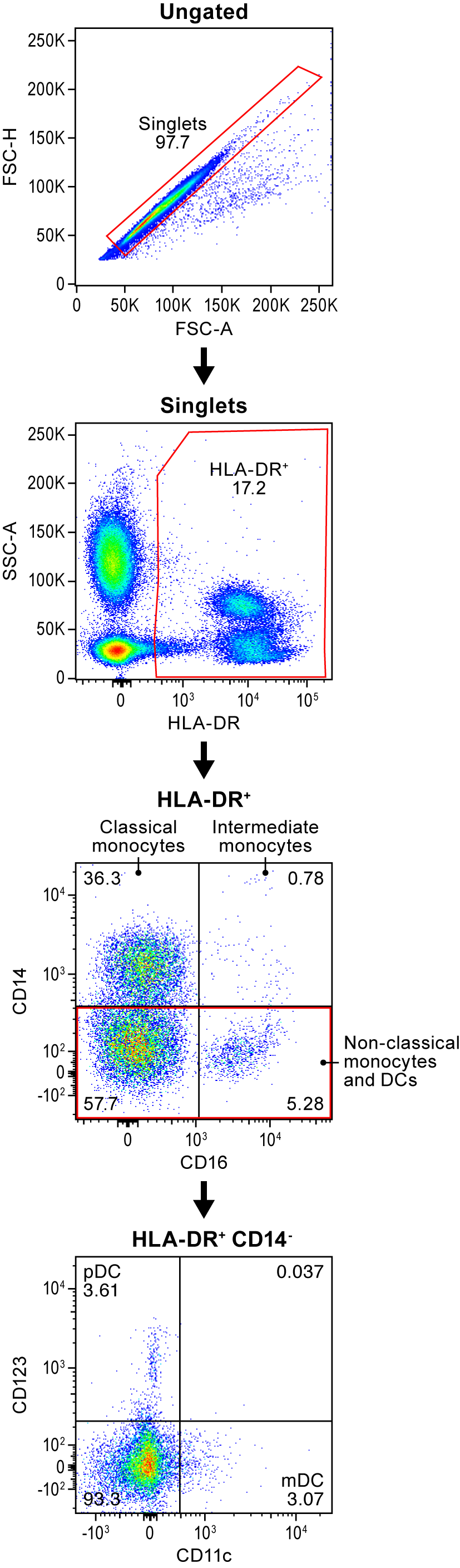

Supplement: S2 Fig — The gating strategy used for differentiating monocyte and dendritic cell populations was the same for all animals and all timepoints. Testing for the presence of NiV antigen was in addition to determinations for the populations defined here. (TIF) [file pntd.0007454.s002.tif]
