# Supplementary material for: Peripheral immune response in the African green monkey model following Nipah-Malaysia virus exposure by intermediate-size particle aerosol
Source: PLoS Negl Trop Dis. 2019 Jun 5;13(6):e0007454. doi: 10.1371/journal.pntd.0007454 (PMC6576798; doi:10.1371/journal.pntd.0007454)
Supplement: S1 Table — Linear regression analysis was performed on the specific cell populations of all animals through the acute phase of disease and additionally on the surviving animal for the complete course of disease. (DOCX) [file pntd.0007454.s003.docx]

S1 Table. Calculated slopes for linear regression analyses
