# Supplementary material for: Peripheral immune response in the African green monkey model following Nipah-Malaysia virus exposure by intermediate-size particle aerosol
Source: PLoS Negl Trop Dis. 2019 Jun 5;13(6):e0007454. doi: 10.1371/journal.pntd.0007454 (PMC6576798; doi:10.1371/journal.pntd.0007454)
Supplement: S2 Table — Target cell surface markers, antibody clones and fluorphores used for the differentiation of peripheral immune cell populations. (DOCX) [file pntd.0007454.s004.docx]

S2 Table. Whole blood panel used for flow cytometry

| Antigen | Clone | Fluorophore |
| --- | --- | --- |
| CD28 | CD28.2 | Brilliant Blue 515 |
| CD14 | M5E2 | Brilliant Violet 650 |
| CD11c | S-HCL-3 | APC |
| CD3 | SP34-2 | Alexa Fluor 700 |
| CD16 | 3G8 | APC-Cy7 |
| Anti-NiV G protein |  | PE |
| CD8α | SK1 | PE/Dazzle594 |
| CD95 | DX2 | PE-Cy5 |
| CXCR3 | CEW33D | PE-Cy7 |
| HLA-DR | L243 | Brilliant Violet 421 |
| CD11b | ICRF44 | Brilliant Violet 510 |
| CD20 | 2H7 | Brilliant Violet 570 |
| CCR6 | 11A9 | Brilliant Violet 605 |
| CD123 | 7G3 | Brilliant Violet 786 |
| CD4 | L200 | Brilliant Violet 711 |
| Ki-67 | B56 | PerCP-Cy5.5 |
