# Supplementary material for: Peripheral immune response in the African green monkey model following Nipah-Malaysia virus exposure by intermediate-size particle aerosol
Source: PLoS Negl Trop Dis. 2019 Jun 5;13(6):e0007454. doi: 10.1371/journal.pntd.0007454 (PMC6576798; doi:10.1371/journal.pntd.0007454)
Supplement: S3 Table — Cell surface markers used to define individual cell populations in the performed analyses. (DOCX) [file pntd.0007454.s005.docx]

S3 Table. Markers used for determining cell type

| Cell Type | Surface Markers |
| --- | --- |
| B Cells | CD3- CD20+ |
| CD4 T Cells | CD3+ CD4+ |
| CD8 T Cells | CD3+ CD8+ |
| Th17 Cells | CD3+ CD4+ CCR6+ CXCR3- |
| Central Memory CD4 Cells | CD3+ CD4+ CD28+ CD95+ |
| Effector Memory CD4 Cells | CD3+ CD4+ CD28- CD95+ |
| Central Memory CD8 Cells | CD3+ CD8+ CD28+ CD95+ |
| Effector Memory CD8 Cells | CD3+ CD8+ CD28- CD95+ |
| Monocytes | CD3- HLA-DR+ CD14+ CD16- |
| Myeloid Dendritic Cells | CD3- CD14- HLA-DR+ CD123- CD11c+ |
| Plasmacytoid Dendritic Cells | CD3- CD14- HLA-DR+ CD123+ CD11c- |
| NK Cells | CD3- CD20- CD14- HLA-DR- NKG2+/CD16+ |
| Basophils | CD45+ HLA-DR- CD123+ |
| Nonclassical Monocytes | CD45+ CD3- CD20- HLA-DR+ CD14- CD16+/- |
